# Six clinical phenotypes with prognostic implications were identified by unsupervised machine learning in children and adolescents with SARS-CoV-2 infection: results from a German nationwide registry

**DOI:** 10.1186/s12931-024-03018-3

**Published:** 2024-10-30

**Authors:** Yanyan Shi, Ralf Strobl, Reinhard Berner, Jakob Armann, Simone Scheithauer, Eva Grill

**Affiliations:** 1 Institute for Medical Information Processing, Biometry and Epidemiology (IBE), Faculty of Medicine, Ludwig-Maximilians-Universität München (LMU Munich), Marchioninistr. 15, 81377 Munich, Germany; 2 Pettenkofer School of Public Health, Munich, Germany; 3 German Center for Vertigo and Balance Disorders, University Hospital, Ludwig-Maximilians- Universität München (LMU Munich), Munich, Germany; 4 Department of Pediatrics, University Hospital and Medical Faculty Carl Gustav Carus, Technische Universität Dresden, Dresden, Germany; 5 Department of Infection Control and Infectious Diseases, University Medical Center Göttingen, Göttingen, Germany

**Keywords:** SARS-CoV-2, Clinical phenotype, Clustering, Machine learning, Prognosis

## Abstract

**Objective:**

Phenotypes are important for patient classification, disease prognostication, and treatment customization. We aimed to identify distinct clinical phenotypes of children and adolescents hospitalized with SARS-CoV-2 infection, and to evaluate their prognostic differences.

**Methods:**

The German Society of Pediatric Infectious Diseases (DGPI) registry is a nationwide, prospective registry for children and adolescents hospitalized with a SARS-CoV-2 infection in Germany. We applied hierarchical clustering for phenotype identification with variables including sex, SARS-CoV-2-related symptoms on admission, pre-existing comorbidities, clinically relevant coinfection, and SARS-CoV-2 risk factors. Outcomes of this study were: discharge status and ICU admission. Discharge status was categorized as: full recovery, residual symptoms, and unfavorable prognosis (including consequential damage that has already been identified as potentially irreversible at the time of discharge and SARS-CoV-2-related death). After acquiring the phenotypes, we evaluated their correlation with discharge status by multinomial logistic regression model, and correlation with ICU admission by binary logistic regression model. We conducted an analogous subgroup analysis for those aged < 1 year (infants) and those aged ⩾ 1 year (non-infants).

**Results:**

The DGPI registry enrolled 6983 patients, through which we identified six distinct phenotypes for children and adolescents with SARS-CoV-2 which can be characterized by their symptom pattern: phenotype A had a range of symptoms, while predominant symptoms of patients with other phenotypes were gastrointestinal (95.9%, B), asymptomatic (95.9%, C), lower respiratory tract (49.8%, D), lower respiratory tract and ear, nose and throat (86.2% and 41.7%, E), and neurological (99.2%, F). Regarding discharge status, patients with D and E phenotype had the highest odds of having residual symptoms (OR: 1.33 [1.11, 1.59] and 1.91 [1.65, 2.21], respectively) and patients with phenotype D were significantly more likely (OR: 4.00 [1.95, 8.19]) to have an unfavorable prognosis. Regarding ICU, patients with phenotype D had higher possibility of ICU admission than staying in normal ward (OR: 4.26 [3.06, 5.98]), compared to patients with phenotype A. The outcomes observed in the infants and non-infants closely resembled those of the entire registered population, except infants did not exhibit typical neurological/neuromuscular phenotypes.

**Conclusions:**

Phenotypes enable pediatric patient stratification by risk and thus assist in personalized patient care. Our findings in SARS-CoV-2-infected population might also be transferable to other infectious diseases.

**Supplementary Information:**

The online version contains supplementary material available at 10.1186/s12931-024-03018-3.

## Introduction

Children and adolescents generally experience mild disease and a better prognosis after severe acute respiratory syndrome coronavirus 2 (SARS‑CoV‑2) infection compared with adults [1, 2]. In some cases, however, severe disease and mortality do occur in the pediatric population as well [3–6]. In Germany, severe disease courses as defined by intensive care unit (ICU) admission occurred in 0.02% of SARS-CoV-2 infections and fatality occurred less than 0.001% during the wild type and the alpha variant [7]. Early stratification of risk groups in order to identify those at highest risk could be beneficial in most appropriate patient care for children and adolescents with SARS‑CoV‑2 infection.

A promising approach to enhance the patient management of children with SARS‑CoV‑2 infection involves the identification of distinctive clinical phenotypes, ideally at the time of hospital admission. Phenotypes reveal how the population can be categorized into homogeneous subgroups with distinct clinical features [8]. In addition to description, phenotypes are important for patient classification, disease prognostication, and treatment customization [8, 9]. Methodologically, clustering is a commonly used unsupervised machine learning method, with which hidden objects, patterns, and groupings were found from untagged data [10]. This approach differs from studies focusing on identifying outcome predictors, which assess the independent predictive association of each variable with the outcome [11]. Clustering has previously been employed in the context of disease phenotyping, such as sepsis [12]. Since the appearance of SARS-CoV-2, it was also applied in identifying the clinical phenotypes of COVID-19 [8, 11, 13]. This approach would allow for the tailoring of standard treatment protocols to accommodate the unique requirements associated with each identified phenotype. While this strategy has been proven effective in optimizing treatment for adults with SARS‑CoV‑2 infection [14], its application in the context of pediatric patients remains to be investigated.

Identification of phenotypes has been utilized in pediatric patients to differentiate severe COVID-19 cases from mild cases and cases with multisystem inflammatory syndrome in Children (MIS-C), also called pediatric multisystem inflammatory syndrome (PIMS), thus enabling more precise treatment according to phenotypes [15]. Our study wanted to adapt this strategy to identify clinical phenotypes with a focus on children and adolescents who tested positive for SARS-CoV-2. Even in the generally low-risk pediatric population, we hypothesized that certain clinical phenotypes representing patient characteristics do exist and that they differ regarding disease severity and an unfavorable prognosis including mortality.

Using data from a German nationwide pediatric registry, we aimed to identify distinct clinical phenotypes of children and adolescents with SARS-CoV-2 infection by clustering, and to assess how the phenotypes differ with regard to disease severity and outcome at discharge.

## Methods

### Data sources

DGPI registry, initiated by the German Society of Pediatric Infectious Diseases (DGPI), is a nationwide, prospective registry for children and adolescents hospitalized with a SARS-CoV-2 infection in Germany. It included patients with laboratory-confirmed SARS-CoV-2 infections who were admitted to pediatric departments and hospitals. A SARS-CoV-2 infection was confirmed if either a reverse transcriptase polymerase chain reaction (RT-PCR) test or, if a nucleic acid based test was not available, an antigen detection rapid diagnostic test (Ag-RDT) for SARS-CoV-2 was reported positive [16]. Details of the DGPI registry has been published before [7, 16]. This registry was approved by the Ethics Committee of the Technische Universität (TU) Dresden (BO-EK-110032020) [16]. Data of patients reported to the DGPI registry from March 2020 to November 2022 were used in the present analysis. This study followed the Strengthening the Reporting of Observational Studies in Epidemiology (STROBE) guideline [17].

### Variables for defining phenotypes

We included variables reported to be associated with severe disease and mortality in children and adolescents with SARS-CoV-2 infection [18–28]. Variables being used for defining phenotypes (Table 1) included sex, SARS-CoV-2-related symptoms at admission, comorbidities at the time of SARS-CoV-2 infection, clinically relevant bacterial or viral coinfection as decided by the pediatricians (Additional file 1, Table S1) at the time of SARS-CoV-2 infection, home oxygen or ventilation therapy prior to the current illness, preterm birth (regardless of the current age), exposure to smoking, immunosuppression, and other SARS-CoV-2 risk factors (Additional file 1, Table S2).

**Table 1:** Description of variables used for defining phenotypes

Domain	Variable	Variable Definition
**Demographics**	Sex	Sex
**Coinfection**	Pulmonary viral coinfection	Clinically relevant coinfection with other respiratory viruses
	Pulmonary bacterial coinfection	Clinically relevant pulmonary coinfection with bacterial infectious agents
	Non-pulmonary bacterial coinfection	Clinically relevant non-pulmonary coinfection with bacterial infectious agents
	Non-pulmonary viral coinfection	Clinically relevant non-pulmonary coinfection with viral infectious agents
**COVID-19 symptoms on admission**	No symptoms (asymptomatic)	No symptoms on admission which were deemed COVID-19 related by corresponding pediatricians of the patients.
	Fever or general symptoms	Fever > 38° Celsius, chills, fatigue, feeling of weakness, weakness to drink / refusal to eat, syncope, dizziness, and other symptoms
	Ear, nose, and throat symptoms	Loss of smell, loss of taste, runny nose, stuffy nose, and other symptoms
	Lower respiratory tract symptoms	Dry cough, productive cough, hemoptysis, retractions of the chest during inhalation, wheezing, tachypnea, shortness of breath, and other symptoms
	Cardiovascular symptoms	Arrhythmias, edema, tachycardia, chest pain, and other symptoms
	Gastrointestinal symptoms	Abdominal pain, diarrhea, nausea, vomiting, loss of appetite, and other symptoms
	Liver symptoms	Jaundice and other symptoms
	Neurological/ neuromuscular symptoms	Disturbance of consciousness or clouding, headache, meningismus, seizure, and other symptoms
	Musculoskeletal Symptoms	Joint pain, muscle pain, inability to walk, and other symptoms
	Other symptoms on admission	Other symptoms on admission which were deemed COVID-19 related by corresponding pediatricians of the patients.
	Unknown admission symptoms	
**Comorbidities at the time of** **SARS-CoV-2 infection**	Respiratory disease	Physician diagnosed comorbidities at the time of the current SARS-CoV-2 infection
	Cardiovascular disease	
	Gastrointestinal tract disease	
	Liver disease	
	Kidney disease	
	Neurological/ neuromuscular disease	
	Psychiatric disease	
	Hematologic disease	
	Oncological disease	
	Organ or bone marrow/stem cell transplantation	
	Autoimmunological disease	
	Congenital immunodeficiency	
	Tracheostoma (prior to COVID-19 infection)	
	Other concomitant disease	
**COVID-19 risk factors**	Home oxygen or ventilation therapy	Oxygen or ventilation therapy before the current SARS-CoV-2 infection
	Preterm birth	The patient was born prematurely
	Exposure to smoking	Both smoking patient and smoking household member were considered to have exposure to smoking
	Immunosuppression	Immunosuppressive medication
	Other COVID-19 risk factors	Other COVID-19 related risk factors (including the newborn patient’s mother was SARS-CoV-2 positive, etc.)

Note: Variable type: sex was binary (male/female), and all other variables were binary (yes/no)

### Outcome variable

Discharge status was the primary outcome of this study. Each patient was assessed at discharge by the pediatricians and was categorized with regard to the admission with a positive SARS-CoV-2 test as: (1) restitutio ad integrum (hereinafter referred to as “full recovery” for easier understanding); (2) residual symptoms that can be considered reversible in the further course of the disease; (3) irreversible consequential damage that has already been identified as potentially irreversible at the time of discharge, such as respiratory failure, heart failure, arrhythmia, renal failure, epilepsy, personality disorder, etc.; (4) transferal to other health facilities; and (5) death, including SARS-CoV-2-related death and non-SARS-CoV-2-related death. In the present analysis, we combined “irreversible consequential damage” and “SARS-CoV-2-related death” as “unfavorable prognosis” due to low case count. Also, patients who were transferred to other health facilities or had non-SARS-CoV-2-related were excluded from further outcome evaluation. Thus, final discharge status was categorized as the three classes: “full recovery”, “residual symptoms”, and “unfavorable prognosis”. ICU stay was the secondary outcome of this study, representing severe disease of COVID-19. It was a binary outcome.

### Statistical analysis

We reported median and interquartile range (IQR) for continuous variables and absolute and relative frequencies for categorical variables.

### Missing values

We assumed the missing values in our dataset were not missing completely at random (MCAR), and checked this assumption by the Little’s MCAR test (R package *naniar*) [29]. The missing information of each variable recorded in the dataset is shown in the footnote of Table 2. Missing values for binary health condition questions (answer: yes/no) were imputed with “no” when physicians skipped the question. The rationale was that non-response indicated a lack of this health condition; this method was also used before [30]. Furthermore, we used random forest (R package *randomForest*) to impute missing values in the variable “sex” as proposed by Breiman [31]. The algorithm starts by imputing missing values with the mode. A Random Forest is fit with this completed data and then used to determine a proximity matrix which is used to update the imputation. The imputed value is the category with the largest average proximity.

### Identifying phenotypes

Variables used for defining phenotypes were described in the above section “*Variables for defining phenotypes”.* We applied hierarchical agglomerative clustering for phenotype identification in the present study, which does not predefine the number of phenotypes. Hierarchical clustering algorithm initially regards each patient as a single cluster and then gradually merges patients most similar to each to new clusters. This process continues until all patients belong to a single cluster. Similarity was computed using Gower’s distance which ranges from 0 to 1, with 0 representing perfect similarity and 1 representing maximum difference [32]. The ongoing merging of clusters was done with respect to minimizing the total within-cluster variance, referred to as Ward´s method [33]. We chose the optimal number of clusters by clinical explanation and the *NbClust* package in R, which offers 30 indices to help decide suitable clustering approach [34]. Hierarchical clustering is usually visualized by dendrogram showing the merging path of each patient (Additional file 1, Figure S1). R (version 4.1.2) and the *cluster* [35] package were used for statistical analysis.

### Prognosis of participants with different phenotypes

We included the entire registered population for comprehensive phenotype identification. Subsequently, we conducted prognostic assessments exclusively on patients with relevant outcomes.

We excluded patients who were transferred to other health facilities and those who died from causes unrelated to SARS-CoV-2 infection because these discharge reasons cannot be considered as unfavorable prognosis regarding a SARS-CoV-2 infection. We used a multinomial logistic regression model to evaluate the associations between distinct phenotypes and discharge status. Since no patient with phenotype B had an unfavorable prognosis, we used two methods of handling phenotype B. For the main model, we excluded patients with phenotype B, and evaluated the associations between other phenotypes and discharge status (including full recovery, residual symptoms, and unfavorable prognosis) in the model. Phenotype A was used as the reference phenotype due to large percentage in the total sample and similarity of symptom pattern to the total sample; full recovery was used as the reference discharge status. Age was included in the model as a confounder. Odds ratios greater than one indicate higher possibility of having residual symptoms or having unfavorable prognosis than achieving full recovery, compared with phenotype A. To investigate the effect of phenotype B, we kept patients with phenotype B but excluded patients with discharge status “unfavorable prognosis”, and evaluated the associations between all phenotypes and discharge status (including full recovery and residual symptoms) with a binary logistic regression model.

We used binary logistic regression to evaluate the associations between distinct phenotypes and ICU stay. Odds ratios greater than one indicate higher possibility of ICU admission than staying in a normal ward, compared with phenotype A. Significance level was set to be 0.05.

### Subgroup analysis

Based on clinical experience, age significantly influences disease severity and clinical outcome in the study population, thus we divided the DGPI registry into infants (age < 1 year) and non-infants (age ⩾ 1 year). We also conducted hierarchical agglomerative clustering for phenotype identification, and applied multinomial logistic regression and binary logistic regression as prognostic assessment for discharge status and ICU stay in these two subgroups. Since only five patients had an unfavorable prognosis in infants, we decided to only compare full recovery and residual symptoms with binary logistic regression in this group. See detailed description of data analysis in Additional file 1, Figure S2.

## Results

### Study participants

The DGPI registry enrolled 6983 patients: 2892 infants and 4091 older children and adolescent. 46.3% of them were female, median age was one year (IQR:0,9). At discharge, 5352 (76.6%) patients were fully recovered, 1526 (21.9%) had residual symptoms, 42 (0.6%) experienced an unfavorable prognosis (including 17 SARS-CoV-2-related deaths), 48 (0.7%) were transferred into another hospital, and 15 (0.2%) had non-SARS-CoV-2-related death. A higher proportion of infants experienced fever or general symptoms, ear, nose and throat (ENT) symptoms, and lower respiratory tract symptoms compared to non-infants, while fewer infants exhibited other symptoms and had comorbidities. See detailed description in Table 2 and Additional file 1, Table S3.

**Table 2:** Characteristics of participants of DGPI registry by discharge status

Variable	All (*n* = 6983)	Discharge Status			
		Full Recovery (*n* = 5352)	Residual Symptoms (*n* = 1526)	Unfavorable Prognosis (*n* = 42)	Transferal/ Non-SARS-CoV-2-related Death (*n* = 63)
**Age (years)**	1 (0,9)	1 (0,8)	2 (0,11)	7 (3,11.7)	9 (3, 13)
**Sex = Female**	3236 (46.3)	2465 (46.1)	717 (47.0)	22 (52.4)	32 (50.8)
**No symptoms (asymptomatic)**	702 (10.1)	677 (12.6)	12 (0.8)	3 (7.1)	10 (15.9)
**Fever or general symptoms**	4818 (69.0)	3620 (67.6)	1145 (75.0)	24 (57.1)	29 (46.0)
**Ear**,** nose**,** and throat symptoms**	1627 (23.3)	1065 (19.9)	550 (36.0)	7 (16.7)	5 (7.9)
**Lower respiratory tract symptoms**	2286 (32.7)	1465 (27.4)	770 (50.5)	27 (64.3)	24 (38.1)
**Cardiovascular symptoms**	226 (3.2)	151 (2.8)	60 (3.9)	9 (21.4)	6 (9.5)
**Gastrointestinal symptoms**	1884 (27.0)	1440 (26.9)	415 (27.2)	8 (19.0)	21 (33.3)
**Liver symptoms**	29 (0.4)	20 (0.4)	7 (0.5)	0 (0.0)	2 (3.2)
**Neurological/ neuromuscular symptoms**	1056 (15.1)	791 (14.8)	240 (15.7)	11 (26.2)	14 (22.2)
**Musculoskeletal Symptoms**	200 (2.9)	131 (2.4)	69 (4.5)	0 (0.0)	0 (0.0)
**Other symptoms on admission**	422 (6.0)	292 (5.5)	116 (7.6)	5 (11.9)	9 (14.3)
**Unknown admission symptoms**	60 (0.9)	43 (0.8)	16 (1.0)	0 (0.0)	1 (1.6)
**Respiratory disease**	295 (4.2)	191 (3.6)	82 (5.4)	8 (19.0)	14 (22.2)
**Cardiovascular disease**	261 (3.7)	184 (3.4)	51 (3.3)	11 (26.2)	15 (23.8)
**Gastrointestinal tract disease**	193 (2.8)	148 (2.8)	34 (2.2)	7 (16.7)	4 (6.3)
**Liver disease**	65 (0.9)	51 (1.0)	11 (0.7)	1 (2.4)	2 (3.2)
**Kidney disease**	145 (2.1)	116 (2.2)	21 (1.4)	2 (4.8)	6 (9.5)
**Neurological/ neuromuscular disease**	445 (6.4)	307 (5.7)	98 (6.4)	20 (47.6)	20 (31.7)
**Psychiatric disease**	111 (1.6)	87 (1.6)	17 (1.1)	2 (4.8)	5 (7.9)
**Hematologic disease**	155 (2.2)	118 (2.2)	30 (2.0)	1 (2.4)	6 (9.5)
**Oncological disease**	106 (1.5)	97 (1.8)	6 (0.4)	0 (0.0)	3 (4.8)
**Organ or bone marrow/stem cell transplantation**	39 (0.6)	33 (0.6)	5 (0.3)	0 (0.0)	1 (1.6)
**Autoimmunological disease**	136 (1.9)	106 (2.0)	21 (1.4)	3 (7.1)	6 (9.5)
**Congenital immunodeficiency**	28 (0.4)	18 (0.3)	8 (0.5)	1 (2.4)	1 (1.6)
**Tracheostoma (prior to current infection)**	18 (0.3)	13 (0.2)	5 (0.3)	0 (0.0)	0 (0.0)
**Other concomitant disease**	965 (13.8)	684 (12.8)	243 (15.9)	17 (40.5)	21 (33.3)
**Pulmonary viral coinfection**	131 (1.9)	72 (1.3)	56 (3.7)	3 (7.1)	0 (0.0)
**Pulmonary bacterial coinfection**	81 (1.2)	38 (0.7)	27 (1.8)	6 (14.3)	10 (15.9)
**Non-pulmonary bacterial coinfection**	331 (4.7)	260 (4.9)	54 (3.5)	8 (19.0)	9 (14.3)
**Non-pulmonary viral coinfection**	136 (1.9)	103 (1.9)	30 (2.0)	1 (2.4)	2 (3.2)
**Home oxygen or ventilation therapy**	111 (1.6)	58 (1.1)	39 (2.6)	7 (16.7)	7 (11.1)
**Preterm birth**	357 (5.1)	270 (5.0)	78 (5.1)	6 (14.3)	3 (4.8)
**Exposure to smoking**	226 (3.2)	149 (2.8)	70 (4.6)	1 (2.4)	6 (9.5)
**Immunsuppression**	149 (2.1)	124 (2.3)	18 (1.2)	2 (4.8)	5 (7.9)
**Other COVID-19 risk factors**	465 (6.7)	313 (5.8)	131 (8.6)	9 (21.4)	12 (19.0)
**Intensive Care Unit stay**	214 (3.1)	107 (2.0)	63 (4.1)	25 (59.5)	19 (30.2)

Note: Number of missing in the above variables: sex (3), respiratory disease (5449), cardiovascular disease (5205), gastrointestinal tract disease (5453), liver disease (5464), kidney disease (5468), neurological/ neuromuscular disease (5443), psychiatric disease (5526), hematologic disease (5462), oncological disease (5466), organ or bone marrow/stem cell transplantation (5472), autoimmunological disease (5472), congenital immunodeficiency (5482), tracheostoma (5476), other concomitant disease (5146), pulmonary viral coinfection (659), pulmonary bacterial coinfection (244), non-pulmonary bacterial coinfection (251), non-pulmonary viral coinfection (1035), home oxygen or ventilation therapy (165), preterm birth (1233), exposure to smoking (1177), immunosuppression (50), other COVID-19 risk factors (1177); other variables did not have missing

### Patient characteristics by phenotypes

Two clusters were proposed as the optimal number of clusters by eight indices in the *NbClust* package, followed by six clusters as the second most frequently proposed optimal number by six indices (Additional file 1, Table S4). To identify the clinically optimal number of phenotypes, we discussed the clinical meaningfulness of the two statistically best solutions, the two phenotypes and the six phenotypes solution, with experienced pediatricians. After this discussion and as a tradeoff between statistical reasoning and better clinical applicability, we decided to report the six phenotype solution as optimal. The six phenotypes varied significantly regarding symptoms on admission, coinfection and SARS-CoV-2 risk factors. Patient characteristics by six phenotypes are shown in Table 3 and Additional file 1, Figure S3.

**Table 3:** Characteristics of participants by phenotypes

Characteristics		Median (IQR) / *n* (%)						
		Total sample (*n* = 6983)	Phenotype A (*n* = 2529)	Phenotype B (*n* = 734)	Phenotype C (*n* = 732)	Phenotype D (*n* = 913)	Phenotype E (*n* = 1460)	Phenotype F (*n* = 615)
**Sex = Female**		3236 (46.3)	1168 (46.2)	378 (51.5)	345 (47.1)	404 (44.2)	665 (45.5)	276 (44.9)
**COVID-19 symptoms on admission**								
	No symptoms (asymptomatic)	702 (10.1)	0 (0.0)	0 (0.0)	702 (95.9)	0 (0.0)	0 (0.0)	0 (0.0)
	General symptoms	4818 (69.0)	2182 (86.3)	457 (62.3)	16 (2.2)	655 (71.7)	1002 (68.6)	506 (82.3)
	Ear, nose, and throat symptoms	1627 (23.3)	675 (26.7)	18 (2.5)	9 (1.2)	197 (21.6)	609 (41.7)	119 (19.3)
	Lower respiratory tract symptoms	2286 (32.7)	414 (16.4)	17 (2.3)	12 (1.6)	455 (49.8)	1259 (86.2)	129 (21.0)
	Cardiovascular symptoms	226 (3.2)	183 (7.2)	0 (0.0)	0 (0.0)	30 (3.3)	7 (0.5)	6 (1.0)
	Gastrointestinal symptoms	1884 (27.0)	437 (17.3)	704 (95.9)	2 (0.3)	269 (29.5)	308 (21.1)	164 (26.7)
	Liver symptoms	29 (0.4)	23 (0.9)	2 (0.3)	1 (0.1)	0 (0.0)	2 (0.1)	1 (0.2)
	Neurological / neuromuscular Symptoms	1056 (15.1)	167 (6.6)	13 (1.8)	8 (1.1)	225 (24.6)	33 (2.3)	610 (99.2)
	Musculoskeletal Symptoms	200 (2.9)	143 (5.7)	4 (0.5)	1 (0.1)	29 (3.2)	6 (0.4)	17 (2.8)
	Other symptoms on admission	422 (6.0)	341 (13.5)	22 (3.0)	1 (0.1)	31 (3.4)	23 (1.6)	4 (0.7)
	Unknown symptoms on admission	60 (0.9)	1 (0.0)	0 (0.0)	1 (0.1)	1 (0.1)	57 (3.9)	0 (0.0)
**Comorbidities at the time of** **COVID-19 infection**								
	Respiratory disease	295 (4.2)	59 (2.3)	8 (1.1)	27 (3.7)	103 (11.3)	96 (6.6)	2 (0.3)
	Cardiovascular disease	261 (3.7)	119 (4.7)	6 (0.8)	27 (3.7)	100 (11.0)	9 (0.6)	0 (0.0)
	Gastrointestinal tract disease	193 (2.8)	50 (2.0)	34 (4.6)	39 (5.3)	53 (5.8)	16 (1.1)	1 (0.2)
	Liver disease	65 (0.9)	16 (0.6)	6 (0.8)	25 (3.4)	18 (2.0)	0 (0.0)	0 (0.0)
	Kidney disease	145 (2.1)	86 (3.4)	1 (0.1)	31 (4.2)	23 (2.5)	1 (0.1)	3 (0.5)
	Neurological/neuromuscular disease	445 (6.4)	55 (2.2)	1 (0.1)	50 (6.8)	312 (34.2)	23 (1.6)	4 (0.7)
	Psychiatric disease	111 (1.6)	11 (0.4)	7 (1.0)	55 (7.5)	22 (2.4)	6 (0.4)	10 (1.6)
	Hematologic disease	155 (2.2)	75 (3.0)	2 (0.3)	25 (3.4)	30 (3.3)	19 (1.3)	4 (0.7)
	Oncological disease	106 (1.5)	61 (2.4)	3 (0.4)	28 (3.8)	8 (0.9)	6 (0.4)	0 (0.0)
	Organ or bone marrow/stem cell transplantation	39 (0.6)	23 (0.9)	0 (0.0)	13 (1.8)	2 (0.2)	0 (0.0)	1 (0.2)
	Autoimmunological disease	136 (1.9)	74 (2.9)	0 (0.0)	27 (3.7)	27 (3.0)	7 (0.5)	1 (0.2)
	Congenital immunodeficiency	28 (0.4)	11 (0.4)	0 (0.0)	3 (0.4)	7 (0.8)	6 (0.4)	1 (0.2)
	Tracheostoma (prior to current infection)	18 (0.3)	1 (0.0)	0 (0.0)	1 (0.1)	14 (1.5)	2 (0.1)	0 (0.0)
	Other concomitant disease	965 (13.8)	152 (6.0)	2 (0.3)	96 (13.1)	679 (74.4)	34 (2.3)	2 (0.3)
**Coinfection**								
	Pulmonary viral infection	131 (1.9)	16 (0.6)	3 (0.4)	4 (0.5)	9 (1.0)	99 (6.8)	0 (0.0)
	Pulmonary bacterial infection	81 (1.2)	16 (0.6)	1 (0.1)	6 (0.8)	30 (3.3)	28 (1.9)	0 (0.0)
	Non-pulmonary bacterial infection	331 (4.7)	232 (9.2)	2 (0.3)	52 (7.1)	31 (3.4)	14 (1.0)	0 (0.0)
	Non-pulmonary viral infection	136 (1.9)	12 (0.5)	92 (12.5)	5 (0.7)	16 (1.8)	9 (0.6)	2 (0.3)
**COVID-19 risk factors**								
	Home oxygen or ventilation therapy before the current disease	111 (1.6)	18 (0.7)	1 (0.1)	7 (1.0)	79 (8.7)	6 (0.4)	0 (0.0)
	preterm infant	357 (5.1)	250 (9.9)	1 (0.1)	45 (6.1)	46 (5.0)	14 (1.0)	1 (0.2)
	Exposure to smoking	226 (3.2)	156 (6.2)	1 (0.1)	31 (4.2)	27 (3.0)	4 (0.3)	7 (1.1)
	Immunosuppression	149 (2.1)	89 (3.5)	2 (0.3)	30 (4.1)	21 (2.3)	5 (0.3)	2 (0.3)
	Other COVID-19 risk factors	465 (6.7)	46 (1.8)	2 (0.3)	36 (4.9)	371 (40.6)	10 (0.7)	0 (0.0)

#### Difference regarding symptoms at admission

Phenotype A had similar symptom pattern as the total sample. Predominant symptoms of patients with other phenotypes were: gastrointestinal symptoms (95.9% in phenotype B), asymptomatic (95.9% in phenotype C), lower respiratory tract symptoms (49.8% in phenotype D), lower respiratory tract symptoms and ENT symptoms (86.2% and 41.7% in phenotype E), and neurological symptoms (99.2%).

#### Difference regarding comorbidities

Patients with phenotype D more frequently had comorbidities - respiratory disease (11.3%), cardiovascular disease (11.0%), gastrointestinal disease (5.8%), liver disease (2.0%), neurological disease (34.2%), psychiatric disease (2.4%), hematological disease (3.3%) and other concomitant diseases (74.4%) than phenotype A, phenotype B, phenotype E and phenotype F (see percentages in Table 3). Patients with phenotype C had similar patterns except for less frequently neurological comorbidity (6.8%), more frequently kidney disease (4.2%), psychiatric disease (7.5%) and oncological disease (3.8%).

#### Difference regarding coinfection

Patients with phenotype A more frequently had non-pulmonary bacterial infection (9.2%, including bloodstream infection, bacterial urinary tract infection/pyelonephritis, and bacterial gastroenteritis); patients with phenotype B more frequently had non-pulmonary viral coinfection (12.5%); patients with phenotype C more frequently had non-pulmonary bacterial infection (7.1%, including bloodstream infection, bacterial arthritis / osteomyelitis, and bacterial urinary tract infection / pyelonephritis); patients with phenotype D more frequently had pulmonary bacterial infection (3.3%, including *Staphylococcus aureus* and *Haemophilus influenzae*); patients with phenotype E more frequently had pulmonary viral infection (6.8%, including respiratory syncytial virus, Influenza virus, human metapneumovirus, human rhinovirus, adenovirus, bocavirus, and enterovirus) and pulmonary bacterial infection (1.9%, including *Streptococcus pneumoniae*, *Haemophilus influenzae*, and Group A *Streptococcus*). See spectrum of coinfection by phenotypes in Additional file 1, Table S1.

#### Difference regarding home oxygen or ventilation therapy and preterm birth

Overall, compared to patients with other phenotypes, patients with phenotype A more frequently had preterm birth (9.9%) and exposure to smoking (6.2%); patients with phenotype C were more likely to receive immunosuppression before current disease (4.1%); patients with phenotype D were more likely to receive home oxygen or ventilation therapy prior to the current disease (8.7%) and to have other SARS-CoV-2 risk factors (40.6%).

#### Difference regarding quarter for hospitalization, SARS-CoV-2 variant, SARS-CoV-2 vaccination, and primary reason for hospitalization

After the phenotypes were identified, we presented the distribution of patients with different phenotypes regarding the quarter for hospitalization, SARS-CoV-2 variant, SARS-CoV-2 vaccination, and primary reason for hospitalization. Phenotypes did not differ significantly in quarter of the year for hospitalization: patients were mostly admitted in the first quarter and least admitted in the third quarter. Additionally, no differences of patients with different phenotypes were observed regarding their infection with different SARS-CoV-2 variants or their vaccination status against SARS-CoV-2. SARS-CoV-2 infection was the primary reason for hospitalization in 3.7% of the patients with phenotype C, 40.5% in phenotype B, 42.0% in phenotype F, and slightly over 50% in other phenotypes (Additional file 1, Table S5).

### Patient characteristics in infants and non-infants

Overall, non-infants and infants exhibited very similar phenotypes to the whole registry. However, phenotype F in infants did not exhibit representative neurological/neuromuscular symptoms at admission as in non-infants (100%) and in the whole registry (99.2%). Instead, phenotype F in infants showed similar attributes as phenotype D whereas with more percentage of patients who had other COVID-19 risk factors (35.6%) (Additional file 1, Table S6 and Table S7).

### Association between phenotypes and clinical outcomes

**Fig. 1:**
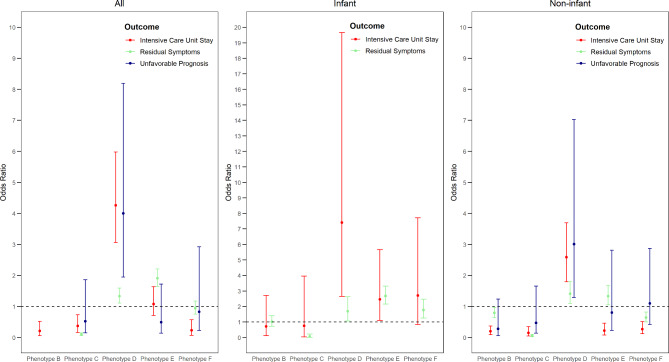
Risk association between phenotypes and clinical outcomes. (1) Phenotype A was the reference phenotype; full recovery was the reference discharge status for residual symptoms and unfavorable prognosis; staying in normal ward was the reference for intensive care unit stay. (2) Odds ratios for residual symptoms and unfavorable prognosis in all registered population and non-infants were estimated with multinomial logistic regression, odds ratios for intensive care unit stay in all groups and odds ratio for residual symptom in infants were estimated with binary logistic regression. (3) Since no patient with phenotype B had an unfavorable prognosis in all registered population, we excluded phenotype B in multinomial logistic regression and evaluated the associations between other phenotypes and discharge status (including full recovery, residual symptoms, and unfavorable prognosis); age was included in the model as a confounder

Figure 1 shows the association between phenotypes and clinical outcomes. Compared to full recovery, patients with phenotype C had a lower risk of having residual symptoms (OR: 0.10 [0.06, 0.15]) than those with phenotype A, whereas patients with D and E phenotype had a higher risk of having residual symptoms (OR: 1.33 [1.11, 1.59] and 1.91 [1.65, 2.21], respectively) than those with phenotype A. Additionally, patients with phenotype D were significantly more likely (OR: 4.00 [1.95, 8.19]) to have an unfavorable prognosis and higher possibility of ICU admission than staying in normal ward (OR: 4.26 [3.06, 5.98]), compared to patients with phenotype A. Patients with phenotype B also had lower risk of having residual symptoms (OR: 0.72 [0.58, 0.89]) than those with phenotype A (Additional file 1, Figure S4).

The outcomes observed in the non-infants with phenotype D and phenotype E closely resembled those of the entire registered population, except for less risk of ICU admission of phenotype E (OR: 0.22 [0.08, 0.46]) than phenotype A. In infants, phenotype D, phenotype E, and phenotype F all had higher risk of having residual symptoms than phenotype A (OR: 1.69 [1.06, 2.62], 2.67 [2.15, 3.31], 1.77 [1.25, 2.47], respectively), and phenotype D and E had higher risk of ICU admission (OR: 7.41 [2.65, 19.65], 2.46 [1.11, 5.67], respectively).

## Discussion

We identified six distinct phenotypes for children and adolescents with SARS-CoV-2 infection by applying an unsupervised machine learning method in a nationwide registry of Germany. We found that patients with phenotype D and phenotype E had higher risk of having residual symptoms than those with phenotype A, and patients with phenotype D also had 4 times risk of having unfavorable prognosis and 4.26 times risk of ICU admission than those phenotype A. Compared to the solution with two phenotypes, we were able to offer insights with a finer granularity into the clinical presentation of children and adolescents with SARS-CoV-2 infection. This stratification also found one specific group which had the highest risk of ICU admission and unfavorable prognosis, thus enabling the most appropriate patient care for them.

Patients with phenotype D primarily exhibited lower respiratory tract symptoms, and were at elevated risk of residual symptoms at discharge, developing unfavorable prognosis, and ICU admission than those with phenotype A. Former studies also reported that independent risk factors for moderate/severe disease involves signs and symptoms such as shortness of breath, rash, seizures, temperature on arrival, chest recessions, and abnormal breath sounds [22, 24]. In addition, we found that patients with phenotype D more frequently had pre-existing comorbidities including respiratory disease, cardiovascular disease, gastrointestinal disease, liver disease, neurological disease, psychiatric disease, hematological disease and other concomitant diseases than other phenotypes. This result is in line with former publications. Geva et al. found one phenotype with frequently pre-existing respiratory conditions needed more invasive or non-invasive mechanical ventilation and had more percentage of deaths, compared to other phenotypes [15].

Patients with phenotype C, primarily asymptomatic, had similar comorbidity patterns as phenotype D, except for less frequently neurological comorbidity, more frequently kidney disease, psychiatric disease and oncological disease. This can be explained by the fact that SARS‑CoV‑2 infection was not the main reason of hospitalization for most of these patients and was found during inpatient stay. Unsurprisingly, patients with this phenotype had lower risk of residual symptoms.

We found that patients with phenotype D more frequently had pulmonary bacterial coinfection and patients with phenotype E more frequently had pulmonary viral coinfection and pulmonary bacterial infection. It has been reported that coinfection with respiratory syncytial virus (RSV) and bacteria was associated with severe illness in infants, and coinfection with RSV was associated with severe illness in COVID-19 patients aged 1 to 4 years [23]. Schober et al. also found that viral coinfection was associated with severe disease of COVID-19 in univariable ordinal logistic regression [19]. Also, patients with phenotype B, characterized mainly by gastrointestinal symptoms, more frequently had non-pulmonary viral infection, and patients with phenotype C, primarily asymptomatic, more frequently had non-pulmonary bacterial infection than patients with other phenotypes. Given that patients with phenotype D had higher risk of both having residual symptoms at discharge and developing unfavorable prognosis than those with phenotype A, we believe pulmonary bacterial coinfection were associated with severe disease of COVID-19 and unfavorable prognosis.

In our study, patients with phenotype D received home oxygen or ventilation therapy before SARS‑CoV‑2 infection than other phenotypes. Farrar et al. also revealed that pre-existing technology dependence requirements including requirement for home oxygen were associated with severe disease [36].

The vast majority of patients with phenotype B had gastrointestinal symptoms. One systematic review showed that gastrointestinal symptoms have been reported in 17.6% of COVID-19 patients [37], and another review reported these manifestations to be more prevalent in children as compared to adults [38]. These symptoms are generally self-limiting, but supportive treatment is needed [38]. This is in line with our study that patients with this phenotype had lower risk of ICU admission. Also, patients with phenotype B more frequently had non-pulmonary viral coinfection than patients with other phenotypes. This coinfection could possibly be viral gastroenteritis, which also needed supportive treatment other than ICU stay.

Patients with phenotype E showed involvement of both lower respiratory tract and ENT. ENT symptoms including dysosmia, dysgeusia, rhinorrhea have been reported in other studies before [39, 40]. One study from Italy showed that loss of taste/smell existed in 3.3% of the participants from primary care at follow-up of 8 to 36 weeks [41]. Thus, it is self-explanatory that patients with phenotype E had higher risk of residual symptoms in our study. Furthermore, we think that patients with phenotype E generally had fewer pre-existing comorbidities than patients with phenotype D was the reason why patients with phenotype E did not show similar prognosis as patients with phenotype D.

It is understandable that patients with phenotype F exhibited typical neurological symptoms, since neurological complications has been documented before in COVID-19 cases [42, 43]. Possible mechanisms of neurological involvement in SARS-CoV-2 infection included direct viral invasion and immune-mediated damage of nervous system [42]. Although it was shown that most neurological symptoms in children and adolescents with SARS-CoV-2 infection were transient and life-threatening conditions were rare [43], severe neurologic manifestations during hospitalization were shown to be associated with new neurocognitive impairments or functional disabilities at hospital discharge [44]. This might explain why patients with phenotype F showed lower risk of ICU admission, but did not exhibit significant difference to the comparator phenotype regarding unfavorable prognosis.

Age was considered as a mortality risk factor for children and adolescents, with an increased risk of death for those younger than two years and those older than 10 years [25, 27, 28]. Our subgroup analysis revealed that infants and non-infants exhibited nearly identical phenotypic characteristics as observed in all registered population. Nevertheless, within infants, phenotype F did not manifest the typical neurological/neuromuscular symptoms observed in all registered population and in non-infants, but rather similar attributes as phenotype D. Furthermore, infants with phenotype D more frequently had preterm birth history. It has also been reported that prematurity was associated with severe COVID-19 [20, 21, 45].

Our work has limitations. Firstly, epidemiological and clinical parameters were used for identifying phenotypes, but laboratory results were not included. Adding laboratory results might result in more refined phenotypes. Secondly, the inclusion of only hospitalized patients necessitates caution when extrapolating the results to the whole infected population. Thirdly, our phenotypes were not validated with an external cohort. Confirmation is warranted regarding whether individuals from other population demonstrate comparable clustering patterns. Lastly, we did not differentiate patients admitted due to SARS-CoV-2 infection and those with incidental positive SARS-CoV-2 test results [46, 47], but we used symptoms on admission which were highly relevant to whether SARS-CoV-2 infection was the primary reason for hospitalization.

## Conclusions

Clustering pediatric patients into phenotypes might help to stratify individuals according to risk and thus assist in tailored patient management. Our findings in SARS-CoV-2-infected population might also be transferable to other infectious diseases.

## Electronic supplementary material

Below is the link to the electronic supplementary material.

Supplementary Material 1

## Data Availability

The dataset supporting the conclusions of this article can be shared upon reasonable requests to Dr. Jakob Armann (Jakob.Armann@ukdd.de).
