# Supplementary material for: Six clinical phenotypes with prognostic implications were identified by unsupervised machine learning in children and adolescents with SARS-CoV-2 infection: results from a German nationwide registry
Source: Respir Res. 2024 Oct 30;25:392. doi: 10.1186/s12931-024-03018-3 (PMC11526611; doi:10.1186/s12931-024-03018-3)
Supplement: Supplementary file 1 — Supplementary Material 1 [file 12931_2024_3018_MOESM1_ESM.pdf]

Six Clinical Phenotypes with Prognostic Implications were identified by Unsupervised Machine Learning in Children and Adolescents with SARS-CoV-2 Infection: Results from a German Nationwide Registry

Table of contents

Table S1 Clinically relevant bacterial or viral coinfection by phenotypes

Table S2 Other SARS-CoV-2 risk factors

Table S3 Characteristics of participants of DGPI registry by age group

Table S4 Number of optimal clusters proposed by *NbClust* package

Table S5 Hospitalization, SARS-CoV-2 variant, and SARS-CoV-2 vaccination status by phenotypes

Table S6 Characteristics of infants by phenotypes

Table S7 Characteristics of non-infants by phenotypes

Figure S1 Dendrogram of hierarchical agglomerative clustering

Figure S2 Data analysis diagram

Figure S3 Heatmap of patient characteristics by clinical phenotypes

Figure S4 Risk association between phenotypes and residual symptoms in all registered population

Table S1 Clinically relevant bacterial or viral coinfection by phenotypes

Characteristics	n (%)						
	**Total sample (n=6983)**	**Phenotype A (n=2529)**	**Phenotype B (n=734)**	**Phenotype C (n=732)**	**Phenotype D (n=913)**	**Phenotype E (n=1460)**	**Phenotype F (n=615)**
Pulmonary viral infection							
Respiratory syncytial virus (RSV)	51 (0.7)	7 (0.3)	1 (0.1)	1 (0.1)	5 (0.5)	37 (2.5)	0 (0.0)
Influenza A or B virus	6 (0.1)	1 (0.0)	0 (0.0)	0 (0.0)	0 (0.0)	5 (0.3)	0 (0.0)
Human metapneumovirus (HPMV)	10 (0.1)	0 (0.0)	0 (0.0)	0 (0.0)	0 (0.0)	10 (0.7)	0 (0.0)
Human rhinovirus (HRV)	36 (0.5)	4 (0.2)	1 (0.1)	3 (0.4)	1 (0.1)	27 (1.8)	0 (0.0)
Adenovirus (respiratory subtypes)	15 (0.2)	1 (0.0)	0 (0.0)	0 (0.0)	0 (0.0)	14 (1.0)	0 (0.0)
Bocavirus	11 (0.2)	1 (0.0)	0 (0.0)	0 (0.0)	1 (0.1)	9 (0.6)	0 (0.0)
Enterovirus (respiratory subtypes)	19 (0.3)	2 (0.1)	2 (0.3)	1 (0.1)	1 (0.1)	13 (0.9)	0 (0.0)
Pulmonary bacterial infection							
Streptococcus pneumoniae	6 (0.1)	1 (0.0)	0 (0.0)	0 (0.0)	1 (0.1)	4 (0.3)	0 (0.0)
Staphylococcus aureus	10 (0.1)	3 (0.1)	0 (0.0)	2 (0.3)	4 (0.4)	1 (0.1)	0 (0.0)
Haemophilus influenzae	11 (0.2)	1 (0.0)	0 (0.0)	0 (0.0)	5 (0.5)	5 (0.3)	0 (0.0)
Group A *Streptococcus*	5 (0.1)	0 (0.0)	0 (0.0)	0 (0.0)	0 (0.0)	5 (0.3)	0 (0.0)
Mycoplasma	6 (0.1)	2 (0.1)	1 (0.1)	0 (0.0)	2 (0.2)	1 (0.1)	0 (0.0)
Non-pulmonary bacterial infection							
Bloodstream infection	34 (0.5)	22 (0.9)	0 (0.0)	5 (0.7)	6 (0.7)	1 (0.1)	0 (0.0)
Bacterial meningitis	3 (0.0)	2 (0.1)	0 (0.0)	0 (0.0)	1 (0.1)	0 (0.0)	0 (0.0)
Bacterial arthritis / osteomyelitis	4 (0.1)	0 (0.0)	0 (0.0)	3 (0.4)	1 (0.1)	0 (0.0)	0 (0.0)
Bacterial urinary tract infection / pyelonephritis	132 (1.9)	103 (4.1)	0 (0.0)	13 (1.8)	10 (1.1)	6 (0.4)	0 (0.0)
Bacterial endocarditis	2 (0.0)	2 (0.1)	0 (0.0)	0 (0.0)	0 (0.0)	0 (0.0)	0 (0.0)
Bacterial gastroenteritis	32 (0.5)	26 (1.0)	2 (0.3)	0 (0.0)	2 (0.2)	2 (0.1)	0 (0.0)

Table S2 Other SARS-CoV-2 risk factors

Variable	n (%)
Other SARS-CoV-2 risk factors	465 (6.7)
The newborn patient’s mother was SARS-CoV-2 positive	9 (0.1)
Not specified	456 (6.6)

Table S3 Characteristics of participants of DGPI registry by age group

Variable	All (n=6983)	Infant (n=2892)	Non-infant (n=4091)
**Age (years, median(IQR))**	1 (0,9)	0 (0,0)	7 (2,13)
**Sex = Female**	3236 (46.3)	1274 (44.1)	1962 (48.0)
**No symptoms (asymptomatic)**	702 (10.1)	165 (5.7)	537 (13.1)
**Fever or general symptoms**	4818 (69.0)	2401 (83.0)	2417 (59.1)
**Ear, nose, and throat symptoms**	1627 (23.3)	771 (26.7)	856 (20.9)
**Lower respiratory tract symptoms**	2286 (32.7)	967 (33.4)	1319 (32.2)
**Cardiovascular symptoms**	226 (3.2)	54 (1.9)	172 (4.2)
**Gastrointestinal symptoms**	1884 (27.0)	631 (21.8)	1253 (30.6)
**Liver symptoms**	29 (0.4)	11 (0.4)	18 (0.4)
**Neurological/ neuromuscular symptoms**	1056 (15.1)	131 (4.5)	925 (22.6)
**Musculoskeletal Symptoms**	200 (2.9)	8 (0.3)	192 (4.7)
**Other symptoms on admission**	422 (6.0)	116 (4.0)	306 (7.5)
**Unknown admission symptoms**	60 (0.9)	16 (0.6)	44 (1.1)
**Respiratory disease**	295 (4.2)	53 (1.8)	242 (5.9)
**Cardiovascular disease**	261 (3.7)	88 (3.0)	173 (4.2)
**Gastrointestinal tract disease**	193 (2.8)	32 (1.1)	161 (3.9)
**Liver disease**	65 (0.9)	25 (0.9)	40 (1.0)
**Kidney disease**	145 (2.1)	37 (1.3)	108 (2.6)
**Neurological/ neuromuscular disease**	445 (6.4)	38 (1.3)	407 (9.9)
**Psychiatric disease**	111 (1.6)	1 (0.0)	110 (2.7)
**Hematologic disease**	155 (2.2)	19 (0.7)	136 (3.3)
**Oncological disease**	106 (1.5)	4 (0.1)	102 (2.5)
**Organ or bone marrow/stem cell transplantation**	39 (0.6)	2 (0.1)	37 (0.9)
**Autoimmunological disease**	136 (1.9)	1 (0.0)	135 (3.3)
**Congenital immunodeficiency**	28 (0.4)	1 (0.0)	27 (0.7)
**Tracheostoma (prior to current infection)**	18 (0.3)	0 (0.0)	18 (0.4)
**Other concomitant disease**	965 (13.8)	349 (12.1)	616 (15.1)
**Pulmonary viral coinfection**	131 (1.9)	62 (2.1)	69 (1.7)
**Pulmonary bacterial coinfection**	81 (1.2)	11 (0.4)	70 (1.7)
**Non-pulmonary bacterial coinfection**	331 (4.7)	121 (4.2)	210 (5.1)
**Non-pulmonary viral coinfection**	136 (1.9)	38 (1.3)	98 (2.4)

**Home oxygen or ventilation therapy**	111 (1.6)	37 (1.3)	74 (1.8)
**Preterm birth**	357 (5.1)	240 (8.3)	117 (2.9)
**Exposure to smoking**	226 (3.2)	69 (2.4)	157 (3.8)
**Immunsuppression**	149 (2.1)	4 (0.1)	145 (3.5)
**Other COVID-19 risk factors**	465 (6.7)	136 (4.7)	329 (8.0)

Table S4 Number of optimal clusters proposed by *NbClust* package

Index	Index Origin	Algorithm for optimal number of clusters	Optimal clusters	Index value
CH	Calinski and Harabasz 1974	Maximum value of the index	2	614.91
DB	Davies and Bouldin 1979	Minimum value of the index	2	1.88
Silhouette	Rousseeuw 1987	Maximum value of the index	2	0.23
Frey	Frey and Van Groenewoud 1972	The cluster level before that index value < 1.00	2	1.28
McClain	McClain and Rao 1975	Minimum value of the index	2	0.18
Dunn	Dunn 1974	Maximum value of the index	2	0.05
SDindex	Halkidi et al. 2000	Minimum value of the index	2	2.87
SDbw	Halkidi and Vazirgiannis 2001	Minimum value of the index	2	0.81
KL	Krzanowski and Lai 1988	Maximum value of the index	6	1.66
Hartigan	Hartigan 1975	Maximum difference between hierarchy levels of the index	6	95.76
TraceW	Milligan and Cooper 1985	Maximum value of absolute second differences between levels of the index	6	159.58
Rubin	Friedman and Rubin 1967	Minimum value of second differences between levels of the index	6	-0.02
Hubert	Hubert and Arabie 1985	Graphical method	6	NA
Dindex	Lebart et al. 2000	Graphical method	6	NA
CCC	Sarle 1983	Maximum value of the index	10	39.65
Scott	Scott and Symons 1971	Maximum difference between hierarchy levels of the index	10	7229.82
Friedman	Friedman and Rubin 1967	Maximum difference between hierarchy levels of the index	10	2.11
Cindex	Hubert and Levin 1976	Minimum value of the index	10	0.16
Ratkowsky	Ratkowsky and Lance 1978	Maximum value of the index	5	0.11
Marriot	Marriot 1971	Max. value of second differences between levels of the index	4	9.43E+78
PtBiserial	Milligan 1980, 1981	Maximum value of the index	4	0.28
TrCovW	Milligan and Cooper 1985	Maximum difference between hierarchy levels of the index	3	50722.28
Ball	Ball and Hall 1965	Maximum difference between hierarchy levels of the index	3	2467.01
Duda	Duda and Hart 1973	Smallest n_c such that index > criticalValue	NA	NA
PseudoT2	Duda and Hart 1973	Smallest n_c such that index < criticalValue	NA	NA
Beale	Beale 1969	n_c such that critical value of the index >= alpha	NA	NA

[REFERENCE]

Malika Charrad, Nadia Ghazzali, Véronique Boiteau, Azam Niknafs. NbClust package for determining the best number of clusters. 2014.

Table S5 Hospitalization, SARS-CoV-2 variant, and SARS-CoV-2 vaccination status by phenotypes

Characteristics	n (%)						
	Total sample (n=6983)	Phenotype A (n=2529)	Phenotype B (n=734)	Phenotype C (n=732)	Phenotype D (n=913)	Phenotype E (n=1460)	Phenotype F (n=615)
Quarter of the year for hospitalization							
Q1	3140 (45.0)	1094 (43.3)	360 (49.0)	347 (47.4)	381 (41.7)	654 (44.8)	304 (49.4)
Q2	1397 (20.0)	537 (21.2)	144 (19.6)	126 (17.2)	177 (19.4)	277 (19.0)	136 (22.1)
Q3	965 (13.8)	374 (14.8)	104 (14.2)	83 (11.3)	120 (13.1)	209 (14.3)	75 (12.2)
Q4	1481 (21.2)	524 (20.7)	126 (17.2)	176 (24.0)	235 (25.7)	320 (21.9)	100 (16.3)
SARS-CoV-2 variant							
Wildtype	10 (0.1)	2 (0.1)	1 (0.1)	3 (0.4)	2 (0.2)	2 (0.1)	0 (0.0)
Alpha	34 (0.5)	10 (0.4)	4 (0.5)	1 (0.1)	7 (0.8)	11 (0.8)	1 (0.2)
Beta	3 (0.0)	1 (0.0)	0 (0.0)	0 (0.0)	0 (0.0)	1 (0.1)	1 (0.2)
Delta	128 (1.8)	41 (1.6)	10 (1.4)	25 (3.4)	23 (2.5)	21 (1.4)	8 (1.3)
Omicron	396 (5.7)	143 (5.7)	29 (4.0)	45 (6.1)	53 (5.8)	84 (5.8)	42 (6.8)
Other SARS-CoV-2 variant	1 (0.0)	0 (0.0)	0 (0.0)	0 (0.0)	1 (0.1)	0 (0.0)	0 (0.0)
Missing	6411 (91.8)	2332 (92.2)	690 (94.0)	658 (89.9)	827 (90.6)	1341 (91.8)	563 (91.5)
SARS-CoV-2 vaccination = YES	183 (2.6)	65 (2.6)	17 (2.3)	18 (2.5)	33 (3.6)	32 (2.2)	18 (2.9)
SARS-CoV-2 infection as the primary reason for hospitalization = YES	3156 (45.2)	1292 (51.1)	297 (40.5)	27 (3.7)	472 (51.7)	810 (55.5)	258 (42.0)
Missing	297 (4.3)	125 (4.9)	28 (3.8)	18 (2.5)	25 (2.7)	87 (6.0)	14 (2.3)

Table S6 Characteristics of infants by phenotypes

Characteristics	n (%)						
	Total sample (n=2892)	Phenotype A (n=1248)	Phenotype B (n=350)	Phenotype C (n=166)	Phenotype D (n=124)	Phenotype E (n=771)	Phenotype F (n=233)
**Sex=Female**	1274 (44.1)	569 (45.6)	153 (43.7)	82 (49.4)	47 (37.9)	333 (43.2)	90 (38.6)
**COVID-19 symptoms on admission**							
No symptoms (asymptomatic)	165 (5.7)	0 (0.0)	0 (0.0)	165 (99.4)	0 (0.0)	0 (0.0)	0 (0.0)
General symptoms	2401 (83.0)	1178 (94.4)	277 (79.1)	0 (0.0)	120 (96.8)	611 (79.2)	215 (92.3)
Ear, nose and throat symptoms	771 (26.7)	298 (23.9)	5 (1.4)	0 (0.0)	45 (36.3)	369 (47.9)	54 (23.2)
Lower respiratory tract symptoms	967 (33.4)	26 (2.1)	53 (15.1)	0 (0.0)	69 (55.6)	717 (93.0)	102 (43.8)
Cardiovascular symptoms	54 (1.9)	20 (1.6)	5 (1.4)	1 (0.6)	7 (5.6)	19 (2.5)	2 (0.9)
Gastrointestinal symptoms	631 (21.8)	95 (7.6)	350 (100.0)	0 (0.0)	14 (11.3)	123 (16.0)	49 (21.0)
Liver symptoms	11 (0.4)	8 (0.6)	2 (0.6)	0 (0.0)	1 (0.8)	0 (0.0)	0 (0.0)
Neurological / neuromuscular Symptoms	131 (4.5)	90 (7.2)	0 (0.0)	0 (0.0)	4 (3.2)	36 (4.7)	1 (0.4)
Musculoskeletal Symptoms	8 (0.3)	4 (0.3)	1 (0.3)	0 (0.0)	1 (0.8)	1 (0.1)	1 (0.4)
Other symptoms on admission	116 (4.0)	86 (6.9)	11 (3.1)	1 (0.6)	2 (1.6)	11 (1.4)	5 (2.1)
Unknown symptoms on admission	16 (0.6)	16 (1.3)	0 (0.0)	0 (0.0)	0 (0.0)	0 (0.0)	0 (0.0)
**Comorbidities at the time of COVID-19 infection**							
Respiratory disease	53 (1.8)	2 (0.2)	4 (1.1)	9 (5.4)	20 (16.1)	13 (1.7)	5 (2.1)
Cardiovascular disease	88 (3.0)	23 (1.8)	2 (0.6)	9 (5.4)	21 (16.9)	21 (2.7)	12 (5.2)
Gastrointestinal tract disease	32 (1.1)	6 (0.5)	3 (0.9)	5 (3.0)	4 (3.2)	10 (1.3)	4 (1.7)
Liver disease	25 (0.9)	7 (0.6)	0 (0.0)	11 (6.6)	4 (3.2)	3 (0.4)	0 (0.0)
Kidney disease	37 (1.3)	17 (1.4)	1 (0.3)	5 (3.0)	7 (5.6)	3 (0.4)	4 (1.7)
Neurological/neuromuscular disease	38 (1.3)	6 (0.5)	2 (0.6)	8 (4.8)	11 (8.9)	7 (0.9)	4 (1.7)
Psychiatric disease	1 (0.0)	0 (0.0)	0 (0.0)	0 (0.0)	0 (0.0)	1 (0.1)	0 (0.0)
Hematologic disease	19 (0.7)	9 (0.7)	1 (0.3)	2 (1.2)	2 (1.6)	3 (0.4)	2 (0.9)
Oncological disease	4 (0.1)	3 (0.2)	0 (0.0)	1 (0.6)	0 (0.0)	0 (0.0)	0 (0.0)
Organ or bone marrow/stem cell transplantation	2 (0.1)	0 (0.0)	0 (0.0)	2 (1.2)	0 (0.0)	0 (0.0)	0 (0.0)

Autoimmunological disease	1 (0.0)	0 (0.0)	0 (0.0)	0 (0.0)	0 (0.0)	0 (0.0)	1 (0.4)
Congenital immunodeficiency	1 (0.0)	1 (0.1)	0 (0.0)	0 (0.0)	0 (0.0)	0 (0.0)	0 (0.0)
Tracheostoma (prior to current infection)	0 (0.0)	0 (0.0)	0 (0.0)	0 (0.0)	0 (0.0)	0 (0.0)	0 (0.0)
Other concomitant disease	349 (12.1)	35 (2.8)	6 (1.7)	32 (19.3)	18 (14.5)	31 (4.0)	227 (97.4)
**Coinfection**							
Pulmonary viral infection	62 (2.1)	5 (0.4)	8 (2.3)	0 (0.0)	0 (0.0)	45 (5.8)	4 (1.7)
Pulmonary bacterial infection	11 (0.4)	1 (0.1)	0 (0.0)	1 (0.6)	1 (0.8)	7 (0.9)	1 (0.4)
Non-pulmonary bacterial infection	121 (4.2)	75 (6.0)	3 (0.9)	13 (7.8)	4 (3.2)	17 (2.2)	9 (3.9)
Non-pulmonary viral infection	38 (1.3)	7 (0.6)	21 (6.0)	1 (0.6)	1 (0.8)	4 (0.5)	4 (1.7)
**COVID-19 risk factors**							
Home oxygen or ventilation therapy before the current disease	37 (1.3)	3 (0.2)	7 (2.0)	4 (2.4)	9 (7.3)	9 (1.2)	5 (2.1)
preterm infant	240 (8.3)	12 (1.0)	30 (8.6)	35 (21.1)	122 (98.4)	24 (3.1)	17 (7.3)
Exposure to smoking	69 (2.4)	39 (3.1)	0 (0.0)	3 (1.8)	1 (0.8)	22 (2.9)	4 (1.7)
Immunosuppression	4 (0.1)	2 (0.2)	0 (0.0)	0 (0.0)	0 (0.0)	0 (0.0)	2 (0.9)
Other COVID-19 risk factors	136 (4.7)	14 (1.1)	2 (0.6)	11 (6.6)	11 (8.9)	15 (1.9)	83 (35.6)

Table S7 Characteristics of non-infants by phenotypes

Characteristics	n (%)						
	Total sample (n=4091)	Phenotype A (n=1522)	Phenotype B (n=720)	Phenotype C (n=507)	Phenotype D (n=371)	Phenotype E (n=439)	Phenotype F (n=532)
**Sex=Female**	1962 (48.0)	746 (49.0)	405 (56.2)	229 (45.2)	159 (42.9)	185 (42.1)	238 (44.7)
**COVID-19 symptoms on admission**							
No symptoms (asymptomatic)	537 (13.1)	30 (2.0)	0 (0.0)	507 (100.0)	0 (0.0)	0 (0.0)	0 (0.0)
General symptoms	2417 (59.1)	1076 (70.7)	399 (55.4)	0 (0.0)	242 (65.2)	289 (65.8)	411 (77.3)
Ear, nose, and throat symptoms	856 (20.9)	224 (14.7)	105 (14.6)	0 (0.0)	96 (25.9)	422 (96.1)	9 (1.7)
Lower respiratory tract symptoms	1319 (32.2)	656 (43.1)	147 (20.4)	0 (0.0)	228 (61.5)	223 (50.8)	65 (12.2)
Cardiovascular symptoms	172 (4.2)	129 (8.5)	3 (0.4)	0 (0.0)	20 (5.4)	13 (3.0)	7 (1.3)
Gastrointestinal symptoms	1253 (30.6)	266 (17.5)	682 (94.7)	0 (0.0)	123 (33.2)	47 (10.7)	135 (25.4)
Liver symptoms	18 (0.4)	8 (0.5)	5 (0.7)	0 (0.0)	1 (0.3)	3 (0.7)	1 (0.2)
Neurological / neuromuscular Symptoms	925 (22.6)	145 (9.5)	46 (6.4)	0 (0.0)	83 (22.4)	119 (27.1)	532 (100.0)
Musculoskeletal Symptoms	192 (4.7)	154 (10.1)	4 (0.6)	0 (0.0)	20 (5.4)	5 (1.1)	9 (1.7)
Other symptoms on admission	306 (7.5)	277 (18.2)	9 (1.2)	0 (0.0)	12 (3.2)	4 (0.9)	4 (0.8)
Unknown symptoms on admission	44 (1.1)	44 (2.9)	0 (0.0)	0 (0.0)	0 (0.0)	0 (0.0)	0 (0.0)
**Comorbidities at the time of COVID-19 infection**							
Respiratory disease	242 (5.9)	165 (10.8)	15 (2.1)	14 (2.8)	41 (11.1)	4 (0.9)	3 (0.6)
Cardiovascular disease	173 (4.2)	99 (6.5)	6 (0.8)	14 (2.8)	39 (10.5)	4 (0.9)	11 (2.1)
Gastrointestinal tract disease	161 (3.9)	60 (3.9)	44 (6.1)	28 (5.5)	22 (5.9)	3 (0.7)	4 (0.8)
Liver disease	40 (1.0)	17 (1.1)	7 (1.0)	5 (1.0)	11 (3.0)	0 (0.0)	0 (0.0)
Kidney disease	108 (2.6)	63 (4.1)	13 (1.8)	18 (3.6)	6 (1.6)	3 (0.7)	5 (0.9)
Neurological/neuromuscular disease	407 (9.9)	163 (10.7)	30 (4.2)	37 (7.3)	75 (20.2)	19 (4.3)	83 (15.6)
Psychiatric disease	110 (2.7)	27 (1.8)	19 (2.6)	33 (6.5)	7 (1.9)	15 (3.4)	9 (1.7)
Hematologic disease	136 (3.3)	71 (4.7)	13 (1.8)	17 (3.4)	20 (5.4)	11 (2.5)	4 (0.8)
Oncological disease	102 (2.5)	91 (6.0)	5 (0.7)	3 (0.6)	1 (0.3)	2 (0.5)	0 (0.0)
Organ or bone marrow/stem cell transplantation	37 (0.9)	32 (2.1)	0 (0.0)	3 (0.6)	0 (0.0)	1 (0.2)	1 (0.2)

Autoimmunological disease	135 (3.3)	84 (5.5)	6 (0.8)	23 (4.5)	19 (5.1)	1 (0.2)	2 (0.4)
Congenital immunodeficiency	27 (0.7)	13 (0.9)	5 (0.7)	1 (0.2)	5 (1.3)	2 (0.5)	1 (0.2)
Tracheostoma (prior to current infection)	18 (0.4)	17 (1.1)	0 (0.0)	1 (0.2)	0 (0.0)	0 (0.0)	0 (0.0)
Other concomitant disease	616 (15.1)	140 (9.2)	39 (5.4)	63 (12.4)	360 (97.0)	11 (2.5)	3 (0.6)
**Coinfection**							
Pulmonary viral infection	69 (1.7)	62 (4.1)	1 (0.1)	3 (0.6)	2 (0.5)	1 (0.2)	0 (0.0)
Pulmonary bacterial infection	70 (1.7)	46 (3.0)	2 (0.3)	5 (1.0)	12 (3.2)	4 (0.9)	1 (0.2)
Non-pulmonary bacterial infection	210 (5.1)	60 (3.9)	81 (11.2)	36 (7.1)	18 (4.9)	15 (3.4)	0 (0.0)
Non-pulmonary viral infection	98 (2.4)	29 (1.9)	52 (7.2)	3 (0.6)	11 (3.0)	2 (0.5)	1 (0.2)
**COVID-19 risk factors**							
Home oxygen or ventilation therapy before the current disease	74 (1.8)	59 (3.9)	1 (0.1)	1 (0.2)	11 (3.0)	1 (0.2)	1 (0.2)
preterm infant	117 (2.9)	88 (5.8)	11 (1.5)	5 (1.0)	7 (1.9)	4 (0.9)	2 (0.4)
Exposure to smoking	157 (3.8)	87 (5.7)	11 (1.5)	22 (4.3)	14 (3.8)	15 (3.4)	8 (1.5)
Immunosuppression	145 (3.5)	123 (8.1)	5 (0.7)	6 (1.2)	7 (1.9)	0 (0.0)	4 (0.8)
Other COVID-19 risk factors	329 (8.0)	43 (2.8)	4 (0.6)	25 (4.9)	245 (66.0)	7 (1.6)	5 (0.9)

Figure S1 Dendrogram of hierarchical agglomerative clustering

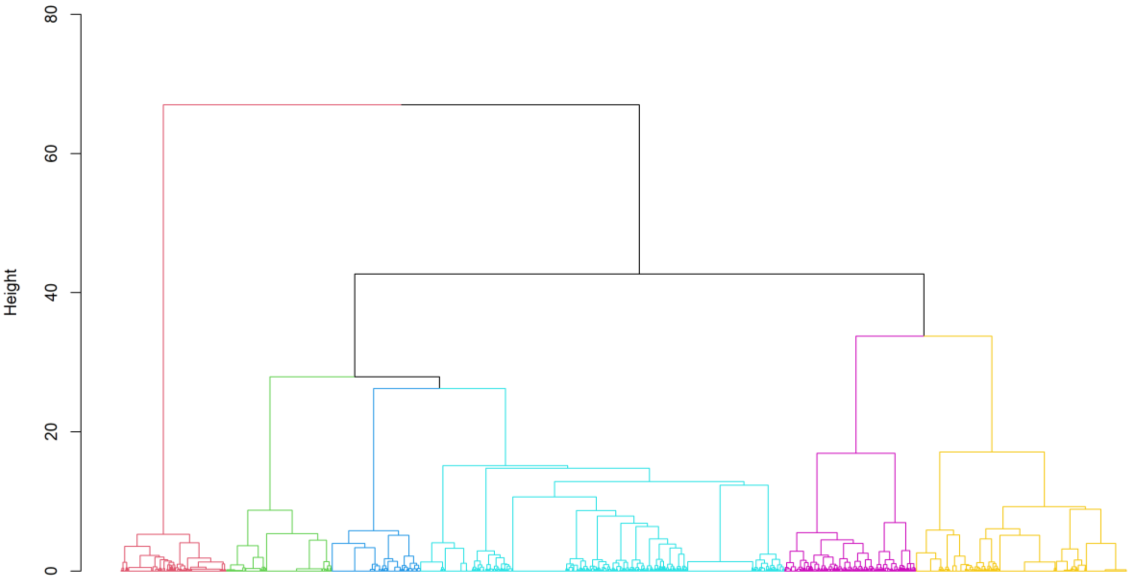

Figure S1 displays the dendrogram resulting from hierarchical clustering, employing Gower' distance and Ward's linkage, utilizing 35 patient characteristics. The y-axis of the dendrogram represents the distance used to cluster the objects ("height"). Initially, each observation is treated as its own cluster, and as the process proceeds in an agglomerative manner, similar objects are grouped together. The height on the y-axis increases as the number of clusters decreases, signifying increased heterogeneity among clusters. Finally, six distinct colors represent the six clusters determined.

[REFERENCE]

Wang M, Flexeder C, Harris CP, et al. Accelerometry-assessed sleep clusters and cardiometabolic risk factors in adolescents. Obesity (Silver Spring). 2024; 32(1): 200-213.

Figure S2 Data analysis diagram

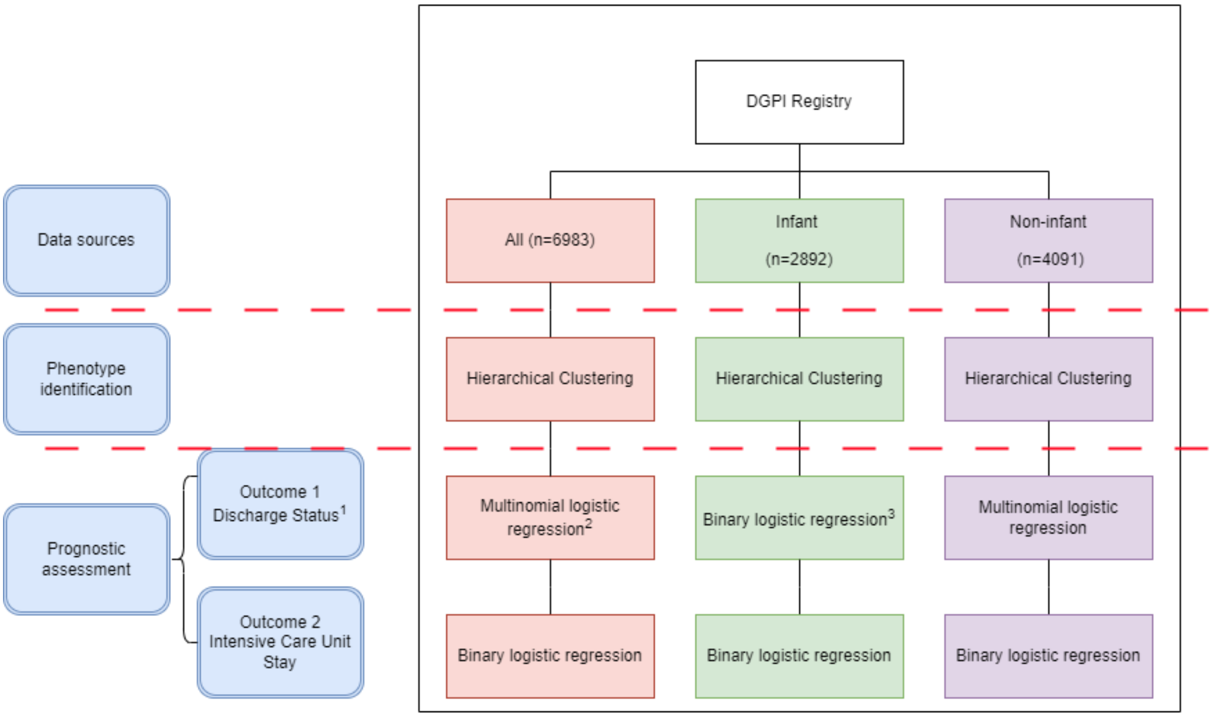

1. Discharge Status included: full recovery, residual symptoms, and unfavorable prognosis; full recovery was used as the reference; patients whose discharge status was transferal or non-SARS-CoV-2-related death were excluded for discharge status assessment. 2. Since no patient with phenotype B had an unfavorable prognosis, we utilized two methods of handling phenotype B. For the main model, we excluded patients with phenotype B, and evaluated the associations between other phenotypes and discharge status (including full recovery, residual symptoms, and unfavorable prognosis) in the model. As a contrast, we excluded patients whose discharge status were "unfavorable prognosis", and evaluated the associations between all phenotypes and discharge status (including full recovery and residual symptoms) with a binary logistic regression model. 3. Since only 5 patients had an unfavorable prognosis in infants, we decided to only compare full recovery and residual symptoms with binary logistic regression in this group.

Figure S3 Heatmap of patient characteristics by clinical phenotypes

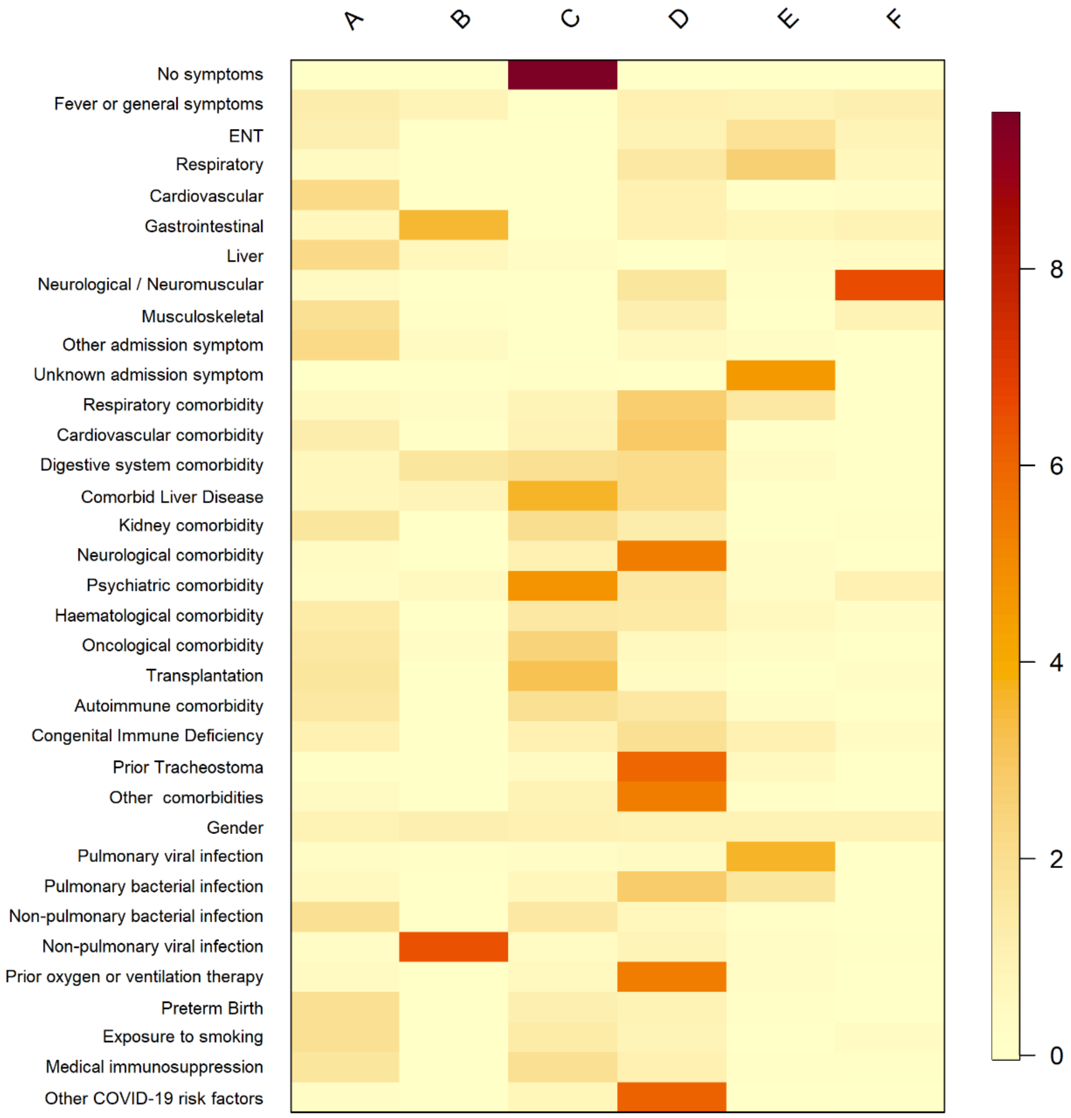

This heatmap shows the difference of percentages of variables defining phenotypes in each phenotype in relation to all the registered population.

Figure S4 Risk association between phenotypes and residual symptoms in all registered population

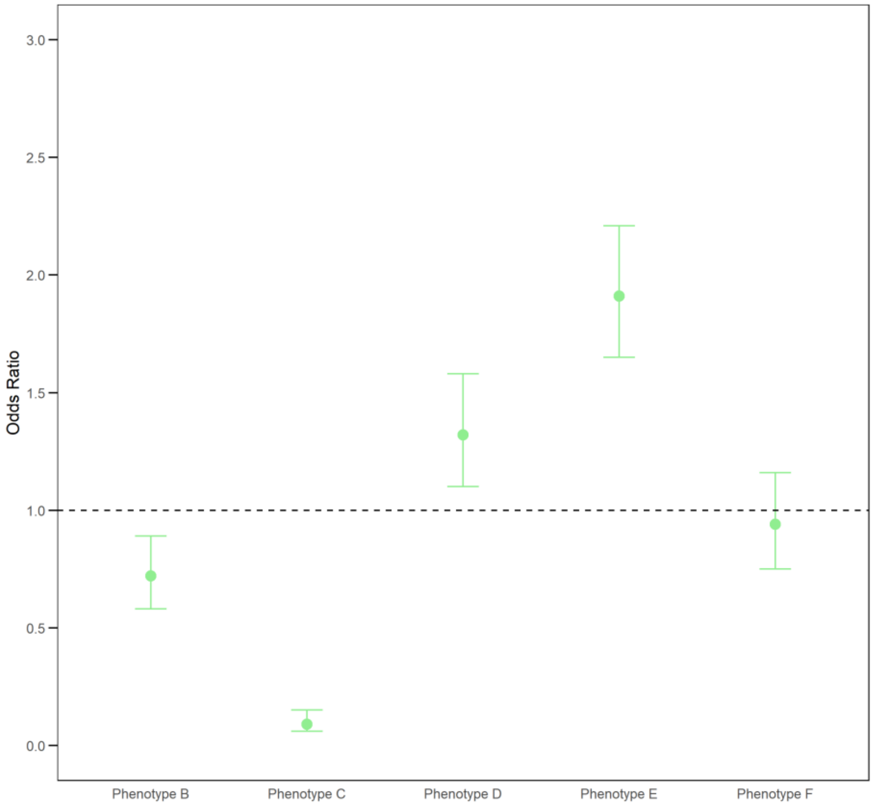

In this model, we excluded patients whose discharge status were “unfavorable prognosis”, and evaluated the associations between all phenotypes and discharge status (including full recovery and residual symptoms) with a binary logistic regression model.
